# Pulmonary Impairment in Tuberculosis Survivors: The Korean National Health and Nutrition Examination Survey 2008-2012

**DOI:** 10.1371/journal.pone.0141230

**Published:** 2015-10-23

**Authors:** Jae-Woo Jung, Jae-Chol Choi, Jong-Wook Shin, Jae-Yeol Kim, Byoung-Whui Choi, In-Won Park

**Affiliations:** Department of Internal medicine, Chung-Ang University College of Medicine, Seoul, Korea; Hospital San Agustín. Aviles. Asturias. Spain, SPAIN

## Abstract

**Objectives:**

Pulmonary tuberculosis (TB) can affect lung function, but studies regarding long-term follow-up in patients with no sequelae on chest X-ray (CXR) have not been performed. We evaluated lung functional impairment and persistent respiratory symptoms in those with prior pulmonary TB and those with prior pulmonary TB with no residual sequelae on CXR, and determined risk factors for airflow obstruction.

**Methods:**

We used data from adults aged ≥ 40 years from the annual Korean National Health and Nutrition Examination Surveys conducted between 2008 and 2012. *P* values for comparisons were adjusted for age, sex, and smoking status.

**Results:**

In total of 14,967 adults, 822 subjects (5.5%) had diagnosed and treated pulmonary TB (mean 29.0 years ago). The FVC% (84.9 vs. 92.6), FEV_1_% (83.4 vs. 92.4), and FEV_1_/FVC% (73.4 vs. 77.9) were significantly decreased in subjects with prior pulmonary TB compared to those without (*p* < 0.001, each). In 12,885 subjects with no sequalae on CXR, those with prior pulmonary TB (296, 2.3%) had significantly lower FEV_1_% (90.9 vs. 93.4, *p* = 0.001) and FEV_1_/FVC% (76.6 vs. 78.4, *p* < 0.001) than those without. Subjects with prior pulmonary TB as well as subjects with no sequalae on CXR were more likely to experience cough and physical activity limitations due to pulmonary symptoms than those without prior pulmonary TB (*p* < 0.001, each). In total subjects, prior pulmonary TB (OR, 2.314; 95% CI, 1.922–2.785), along with age, male, asthma, and smoking mount was risk factor for airflow obstruction. In subjects with prior pulmonary tuberculosis, inactive TB lesion on chest x-ray (OR, 2.300; 95% CI, 1.606–3.294) were risk factors of airflow obstruction.

**Conclusion:**

In addition to subjects with inactive TB lesion on CXR, subjects with no sequelae on CXR can show impaired pulmonary function and respiratory symptoms. Prior TB is a risk factor for airflow obstruction and that the risk is more important when they have inactive lesions on chest X-ray. Hence, the patients with treated TB should need to have regular follow-up of lung function and stop smoking for early detection and prevention of the chronic airway disease.

## Introduction

Tuberculosis (TB) is a distressing disease, as its prevalence and associated mortality continue to increase. In 2013, the prevalence of tuberculosis was 11.9 million, and the number of tuberculosis related deaths was 1.4 million [1]. TB is an infectious disease caused by *Mycobacterium tuberculosis* and its early detection is an effective method for preventing its spread. However, unlike other infectious diseases, TB is a chronic disease; therefore, it causes structural damage or vascular compromise, frequently resulting in acute and subacute complications [2]. In addition, TB induces long-term anatomic alterations of the involved organs and subsequently leads to chronic complications. In particular, TB leads to aspergilloma within residual TB cavities, bronchiectasis, and impaired lung function, which causes long-term morbidity and mortality, as well as a significant burden of medical cost [3].

Numerous studies have reported that chronic airflow obstruction is a sequela to pulmonary TB [4–8]. A previous study on chest X-rays (CXR) indicated that inactive TB lesions are associated with impaired pulmonary function [5]. Furthermore, a cohort study reported that pulmonary function deteriorates a few months after TB medication [7]. However, these studies were limited by small sample size, short-term observation, or inclusion of prior TB cases only assessed by CXR.

In Korea, the prevalence of active TB on CXR decreased from 5,168 in 100,000 in 1965 to 767 in 100,000 in 1995 [9]. According to the 2012 Global Tuberculosis Report from the World Health Organization, the TB incidence in Korea was slightly lower than the global TB incidence (100 vs. 125 in 100,000, respectively), which corresponds to an intermediate national burden of TB [10].

Therefore, we evaluated impaired pulmonary function and the presence of persistent respiratory symptoms and also identified risk factors for long-term pulmonary function impairment in subjects with diagnosed prior pulmonary TB. Data was analyzed from five years of data from the Korean National Health and Nutrition Examination Survey (Korean NHANES), a large-scale, nationwide representative survey. In addition, we investigated if subjects with history of prior pulmonary TB without CXR sequelae were associated with impaired pulmonary function.

## Materials and Methods

### 1. Study subjects

We collected five years of data from the Korean NHANES (2008–2012). This study protocol was approved by the Institutional Review Board of the Korea Centers for Disease Control and Prevention (approval no. 2008-04EXP-01-C, 2009-01CON-03-2C, 2010-02CON-21-C, 2011-02CON-06-C, and 2012-01EXP-01-2C). Written informed consent was obtained from all participants. The Korean NHANES is a population-based nationwide cross-sectional surveillance system conducted annually by the Korea Center for Disease Control and Prevention [5,11]. This survey is a nationwide representative study using a stratified, multistage probability sampling design for the selection of household units. The participants completed questionnaires that included a health interview survey, a health behavior survey, and a nutrition survey and participants also underwent a health examination survey including CXR, biochemical assay and pulmonary function test.

Of the total 45,811 subjects, 14,967 adults aged ≥ 40 years were included in the study. Age, sex, smoking status, and history of pulmonary TB, or asthma diagnosed by a doctor were assessed through a questionnaire. Questions about age and treatment institution of subjects with prior pulmonary TB were also included in the questionnaire. Questions about respiratory symptoms, physical activity limitations due to pulmonary symptoms and the visual analogue scale to assess the global quality of life were used. Visual analogue scale is ranked between 0 ("worst imaginable health state") and 100 ("best imaginable health state"). The participants indicated where they perceive their present state of health to lie to this domain range [12].

### 2. Pulmonary function tests

Pulmonary function tests were performed to each subjects (14,967 adults) using a dry rolling-seal spirometer (Vmax-2130, Sensor-Medics, Yorba Linda, CA, USA). Spirometry was conducted by well-trained pulmonary laboratory technicians. The test was terminated if the curve obtained from 3 measurements of pulmonary function was appropriate or if the test results met repeatability criteria. Otherwise, the test was conducted up to 8 times and until the subjects were no longer able to tolerate the test. Appropriateness curves were based on the 1994 recommendation of the American Thoracic Surgery [13]. Repeatability was achieved if differences in forced vital capacity (FVC) and forced expiratory volume in 1 second (FEV_1_) were less than 150 mL between the greatest and second greatest values. The nurses subsequently forwarded the contents of the quality control tests to the technicians each week. The normal values of FEV_1_ and FVC were based on pulmonary function data from Korean nonsmokers with normal CXR [13]. We measured FEV_1_, FVC, forced expiratory flow 25–75% (FEF_25-75%_), forced expiratory volume in 6 seconds (FEV_6_), and peak expiratory flow (PEF) to evaluate pulmonary function. Airflow obstruction and chronic obstructive pulmonary disease (COPD) was defined as FEV_1_/FVC% < 70 according to the WHO Global Initiative for Chronic Obstructive Lung Disease (GOLD) [14]. In COPD, mild COPD is defined FEV_1_% ≥ 80, moderate, 50 ≤ FEV_1_% < 80, severe, 30 ≤ FEV_1_% < 50 and very severe, FEV_1_% < 30 [14].

### 3. Chest radiography

CXR were taken with DigiRAD-PG (Sitec Medical Co., Ltd, Kimpo-si, Gyeonggi-do, Korea) installed on the examination vehicle. Two independent radiologists read transmitted CXR according to the standard criteria for reporting radiological abnormalities. By using CXR, an inactive TB lesion was defined as a lesion with discrete linear or reticulofibrotic opacities with or without calcification that was confined to the upper lobes, while the other lobes were intact [15]. Any discrepancies among the 2 radiologists were resolved through a consensus conference with a third radiologist.

### 4. Statistical analysis

All statistical analyses were performed using SPSS version 16.0 (SPSS Inc., Chicago, IL, USA). Continuous variables were expressed as the mean and standard deviation (SD), and categorical variables were presented as numbers and percentages. Comparisons of age, sex, and smoking history were made using the Chi-square and Student’s *t* test. *P* values for comparisons of continuous variables and categorical variables between individual groups were all adjusted for age, sex, and smoking amount. Multivariate logistic regression was used to determine risk factors for pulmonary function deterioration. A *p* value of < 0.05 was considered significant.

## Results

### 1. Clinical characteristics of the study subjects

This study included a total of 14,967 adults aged ≥ 40 years. There were 6,486 males (43.3%), and the mean age of the subjects was 57.1 ± 10.9 years (Table 1). There were 822 subjects (5.5%) diagnosed with TB who received treatment by physicians; the mean age of the subjects at the time of diagnosis and the time of TB diagnosis were 30.6 ± 14.3 years and 29.0 ± 13.7 years ago, respectively. Inactive TB lesions on CXR were found in 1,178 (7.9%) of all subjects.

**Table 1:** Clinical characteristics of the study population.

	N = 14,967
Age (years)	57.1 ± 10.9
Male sex	6,486 (43.3%)
Smoking status	
Current smoker	2,828 (18.9%)
Ex-smoker	3,292 (22.0%)
Nonsmoker	8,823 (59.0%)
Smoking status (pack-year)	9.6 ± 16.9
Prior pulmonary tuberculosis	822 (5.5%)
Age of diagnosis (years)	30.6 ± 14.3
Timing of diagnosis (years ago)	29.0 ± 13.7
Medical institution for treatment	
Hospital	233 (28.3%)
Public health center	183 (22.3%)
Other	26 (3.2%)
No answer	380 (46.2%)
Pulmonary function tests	
FVC %	92.2 ± 12.1
FVC (mL)	3,415.1 ± 850.5
FEV_1_%	92.2 ± 13.8
FEV_1_ (mL)	2,645.4 ± 680.6
FEV_1_/FVC %	77.7 ± 7.8
FEV_6_ (mL)	3,332.00 ± 808.36
FEF_25-75%_ (mL/sec)	3,631.51 ± 2,063.75
PEF (mL/sec)	7,050.90 ± 1,916.25
COPD	1,978 (13.2%)
Mild	899 (6.0%)
Moderate	978 (6.5%)
Severe	90 (0.6%)
Very severe	11 (0.1%)
Chest X-ray	
Normal X-ray	12,885 (86.1%)
Inactive tuberculosis lesion	1,178 (7.9%)
Cardiac disease	112 (0.7%)
Other lung diseases	792 (5.3%)

Data are means ± SD or percentages.

FVC: forced vital capacity; FEV_1_: forced expiratory volume in 1 second; FEV_6_: forced expiratory volume in 6 seconds; FEF_25-75%_: forced expiratory flow 25–75%; PEF: peak expiratory flow; COPD: chronic obstructive pulmonary disease.

### 2. Comparison according to the presence of prior pulmonary TB

**Fig 1:**
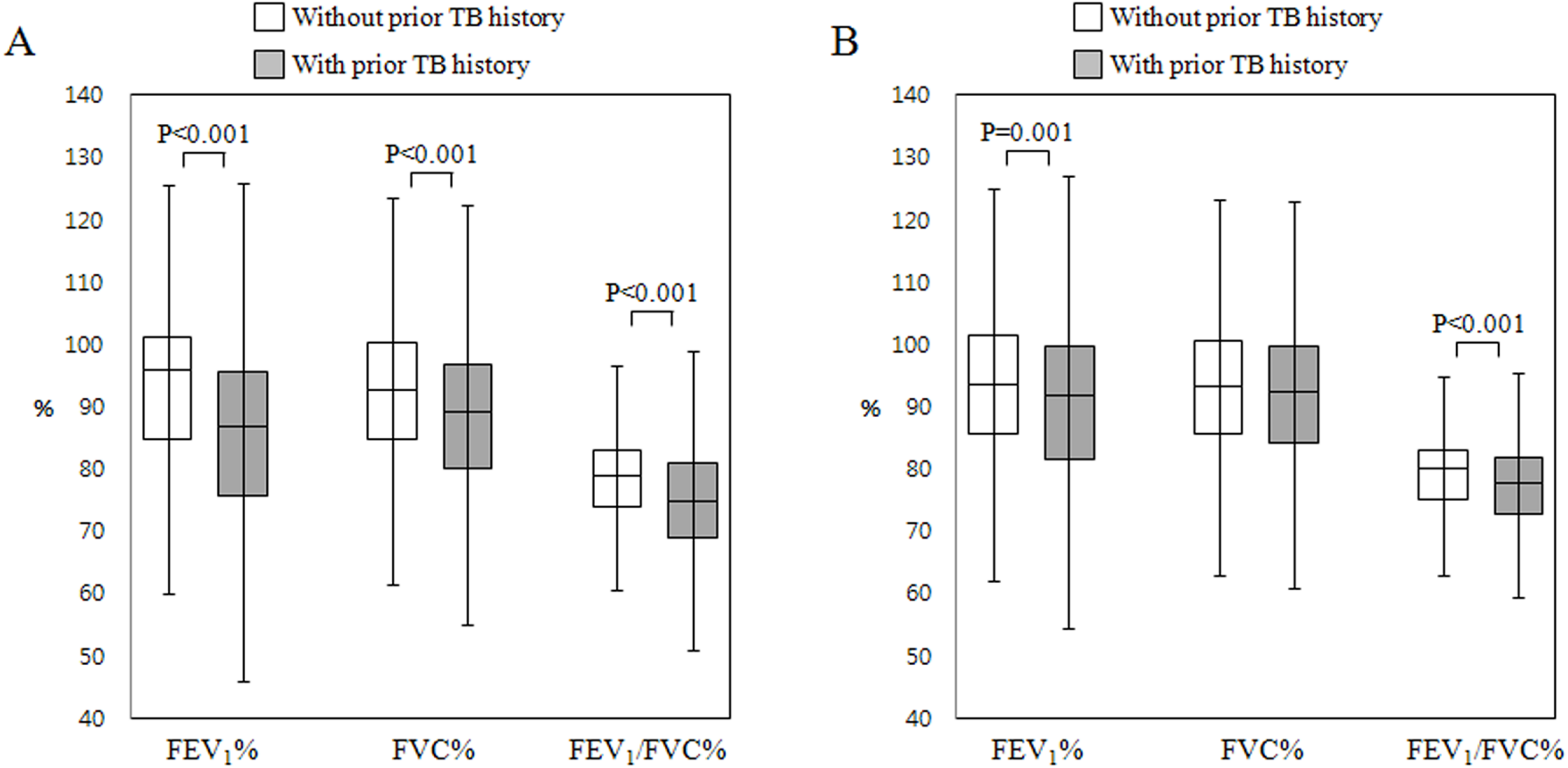
Lung functions according to the presence of prior pulmonary tuberculosis. (A) In all study subjects; (B) In subjects with normal chest X-rays. FVC: forced vital capacity; FEV1: forced expiratory volume in 1 second.

Clinical characteristics were assessed in the entire study population according to TB history of physician-diagnosed prior pulmonary TB (Table 2). Subjects with prior pulmonary TB were older than those without prior pulmonary TB (*p* < 0.001) and showed a higher proportion of males as well (*p* < 0.001). The total amount of smoking was higher in subjects with prior pulmonary TB than in those without prior pulmonary TB (12.0 ± 19.0 pack-years vs. 9.5 ± 16.8 pack-years, *p* < 0.001). In subjects with prior pulmonary TB, the proportion of those who showed inactive TB lesions on CXR was 451 subjects (54.9%), and that of normal CXR was 296 subjects (36.0%). The remained 75 subjects (9.1%) showed cardiac disease and other lung diseases. FEV_1_% (84.9 ± 17.2 vs. 92.6 ± 13.5), FVC% (88.4 ± 13.5 vs. 92.4 ± 12.0), and FEV_1_/FVC% (73.4 ± 10.2 vs. 77.9 ± 7.5) were significantly decreased in subjects with prior pulmonary TB, as compared to those without prior pulmonary TB (*p* < 0.001 for each, Fig 1A). In addition, FEV_6_, FEF_25-75%_, and PEF were further decreased in subjects with prior pulmonary TB (*p* < 0.001). Subjects with prior pulmonary TB showed a higher proportion of COPD than those without prior pulmonary TB (29.1% vs. 12.3%, *p* < 0.001). When looked into each stage of COPD, subjects with prior pulmonary TB showed a higher proportion than those without prior pulmonary TB in every stage (*p* < 0.001 for each).

**Table 2:** Comparison between subjects with or without prior pulmonary tuberculosis.

	Prior pulmonary tuberculosis (-) n = 14,145 (94.5%)	Prior pulmonary tuberculosis (+) n = 822 (5.5%)	*p* value
Age (years)	56.9 ± 10.9	59.5 ± 10.9	< 0.001
Male sex	6,048 (42.8%)	438 (53.3%)	< 0.001
Smoking status			< 0.001
Current smoker	2,679 (19.0%)	149 (18.2%)	
Ex-smoker	3,039 (21.5%)	253 (30.9%)	
Nonsmoker	8,405 (59.5%)	418 (51.0%)	
Smoking status (pack-year)	9.45 ± 16.81	11.95 ± 18.61	< 0.001
Asthma*	438 (3.1%)	57 (6.9%)	
Pulmonary function tests*			< 0.001
FVC %	92.4 ± 12.0	88.4 ± 13.5	< 0.001
FVC (liters)	3.4 ± 0.9	3.4 ± 1.0	< 0.001
FEV_1_%	92.6 ± 13.5	84.9 ± 17.2	
FEV_1_ (liters)	2.7 ± 0.7	2.5 ± 0.7	< 0.001
FEV_1_/FVC %	77.9 ± 7.5	73.4 ± 10.2	< 0.001
FEV_6_ (liters)	3.3 ± 0.8	3.2 ± 0.9	< 0.001
FEF_25-75%_ (liters/sec)	3.7 ± 2.1	3.1 ± 2.1	< 0.001
PEF (liters/sec)	7.1 ± 1.9	6.6 ± 2.1	< 0.001
COPD*	1,739 (12.3%)	239 (29.1%)	< 0.001
Mild	820 (5.8%)	79 (9.6%)	< 0.001
Moderate	847 (6.0%)	131 (15.9%)	< 0.001
Severe	67 (0.5%)	23 (2.8%)	< 0.001
Very severe	5 (0.04%)	6 (0.7%)	< 0.001

Data are means ± SD or percentages.

FVC: forced vital capacity; FEV_1_: forced expiratory volume in 1 second; FEV_6_: forced expiratory volume in 6 seconds; FEF_25-75%_: forced expiratory flow 25–75%; PEF: peak expiratory flow; COPD: chronic obstructive pulmonary disease.

^*^ *p* values were adjusted for age, sex, and smoking status.

In 8,823 never smoker (1,014 males and 7,809 females), FEV_1_% (88.5 ± 13.6 vs. 93.2 ± 11.9), FVC% (86.2 ± 17.0 vs. 94.5 ± 13.2), and FEV_1_/FVC% (76.0 ± 8.3 vs. 79.6 ± 6.1) were significantly decreased in 418 subjects with prior pulmonary TB, as compared to 8,405 subjects without prior pulmonary TB (*p* < 0.001 for each).

### 3. Comparison of subjects with normal chest X-rays according to the presence of prior pulmonary TB

Of all subjects, 12,885 (86.1%) showed normal CXR (Table 3). Among these subjects, FEV_1_% (90.9 ± 13.9 vs. 93.4 ± 12.8, *p* = 0.001), FEV_1_/FVC % (76.6 ± 7.4 vs. 78.4 ± 7.0, *p* < 0.001), and PEF (7.0 ± 1.9 vs. 7.2 ± 1.9, *p* = 0.023) were significantly decreased in those with prior pulmonary TB than in those without prior pulmonary TB (Fig 1B). The proportion of COPD was higher in subjects with prior pulmonary TB than those without prior pulmonary TB (15.5% vs. 10.4%, *p* = 0.004). In moderate COPD, subjects with prior pulmonary TB showed a higher proportion than those without prior pulmonary TB (8.4% vs. 4.8%, *p* = 0.004).

**Table 3:** Comparison between subjects with or without prior pulmonary tuberculosis with normal chest x-rays.

	Prior pulmonary tuberculosis (-) n = 12,589 (97.7%)	Prior pulmonary tuberculosis (+) n = 296 (2.3%)	*p* value
Age (years)	56.0 ± 10.6	57.4 ± 10.9	0.048
Male sex	5,261 (41.6%)	136 (45.9%)	0.130
Smoking status			0.030
Current smoker	2,361 (18.8%)	44 (14.9%)	
Ex-smoker	2,604 (20.7%)	78 (26.4%)	
Nonsmoker	7,605 (60.5%)	173 (58.6%)	
Smoking status (pack-year)	9.0 ± 16.1	9.3 ± 15.8	0.702
Asthma*	364 (2.9%)	17 (5.7%)	0.009
Pulmonary function tests*			
FVC %	93.1 ± 11.6	92.1 ± 12.1	0.369
FVC (liters)	3.4 ± 0.8	3.5 ± 0.9	0.226
FEV_1_%	93.4 ± 12.8	90.9 ± 13.9	0.001
FEV_1_ (liters)	2.7 ± 0.7	2.7 ± 0.7	0.331
FEV_1_/FVC %	78.4 ± 7.0	76.6 ± 7.4	< 0.001
FEV_6_ (liters)	3.4 ± 0.8	3.4 ± 0.9	0.328
FEF_25-75%_ (liters/sec)	3.7 ± 2.1	3.6 ± 2.2	0.628
PEF (liters/sec)	7.2 ± 1.9	7.0 ± 1.9	0.023
COPD*	1,305 (10.4%)	46 (15.5%)	0.004
Mild	670 (5.3%)	19 (6.4%)	0.407
Moderate	607 (4.8%)	25 (8.4%)	0.004
Severe	28 (0.2%)	2 (0.7%)	0.151
Very severe	0	0	-

Data are means ± SD or percentages.

FVC: forced vital capacity; FEV_1_: forced expiratory volume in 1 second; FEV_6_: forced expiratory volume in 6 seconds; FEF_25-75%_: forced expiratory flow 25–75%; PEF: peak expiratory flow; COPD: chronic obstructive pulmonary disease.

*p values were adjusted for age, sex, and smoking status.

In 7,778 never smoker with normal CXR (848 males and 6,930 females), FEV_1_% (92.2 ± 14.6 vs. 95.2 ± 12.6, *p* = 0.008), and FEV_1_/FVC% (78.4 ± 6.5 vs. 80.0 ± 5.8, *p* = 0.002) were significantly decreased in 173 subjects with prior pulmonary TB compared to 7,605 subjects without prior pulmonary TB.

### 4. Frequency of respiratory symptoms and quality of life according to the presence of prior pulmonary TB

**Fig 2:**
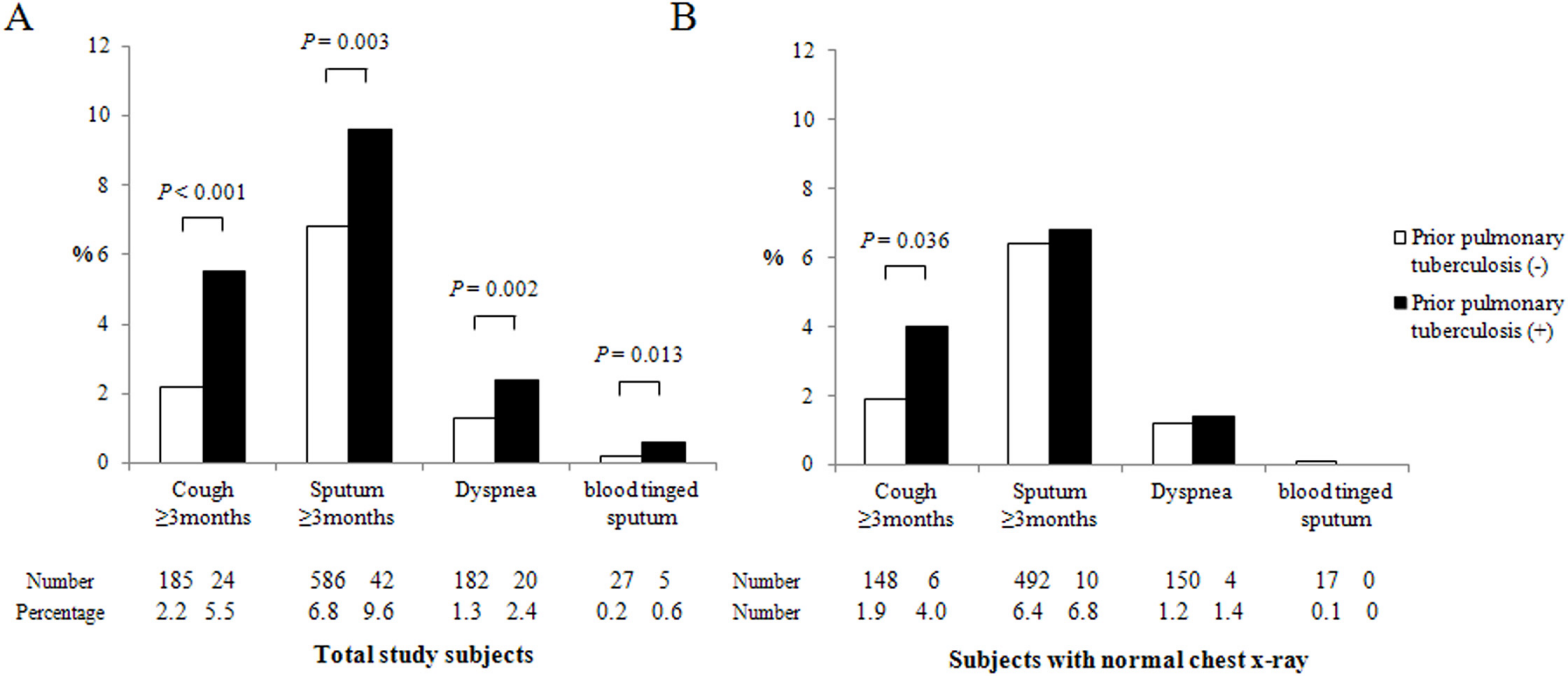
Respiratory symptoms according to the presence of prior pulmonary tuberculosis. (A) In all study subjects; (B) In subjects with normal chest X-rays. *p* values were adjusted for age, sex, and smoking status.

Subjects with prior pulmonary TB more frequently had cough of three or more months during last year (5.5% vs. 2.2%, *p* < 0.001), sputum production of three or more months during last year (9.6% vs. 6.8%, *p* = 0.003), dyspnea (2.4% vs. 1.3%, *p* = 0.002), and blood-tinged sputum (0.6% vs. 0.2%, *p* = 0.013) than those without prior pulmonary TB (Fig 2A). Among subjects with normal CXR, those with prior pulmonary TB more frequently had cough of three or more months during last year than those without prior pulmonary TB (4.0% vs. 1.9%, *p* < 0.001, Fig 2B).

Subjects with prior pulmonary TB more frequently had physical activity limitations due to pulmonary symptoms, as compared to those without prior pulmonary TB in total patients (3.2% vs. 0.6%, *p* < 0.001) and in subjects with normal CXR (1.4% vs. 0.4%, *p* = 0.041).

Scores of visual analogue scale, indicator of quality of life were significantly lower in subjects with prior pulmonary TB than in those without prior pulmonary TB (72.0 ± 18.0 vs. 73.7 ± 18.3, *p* = 0.008). However, in subjects with normal CXR, there was no different in scores of visual analogue scale according to presence of prior pulmonary TB (*p* > 0.05).

### 5. Risk factors for airflow obstruction in total subjects and in subjects with prior pulmonary tuberculosis

Multivariate regression analysis was performed to identify risk factors for airflow obstruction (Table 4). In total subjects, airflow obstruction was significantly associated with age (OR, 1.089; 95% CI, 1.083–1.095), male (OR, 3.368; 05% CI, 2.946–3.851), asthma (OR. 5.014; 95% CI, 4.022–6.250), smoking amount (OR, 1.017; 95% CI, 1.014–1.020), and prior pulmonary TB (OR, 2.314; 95% CI, 1.922–2.785). Prior pulmonary TB was risk factor of FEV_1_% < 80 (OR, 2.691; 95% CI, 2.194–3.301), FEV_1_% < 50 (OR, 4.407; 95% CI, 2.723–7.131) and FEV_1_% < 30 (OR, 8.879; 95% CI, 2.502–31.59) combined with airflow obstruction. In subjects with normal chest x-rays, prior pulmonary TB was risk factor of FEV_1_% < 80 combined with airflow obstruction (OR, 1.589; 95% CI, 1.034–1.023).

**Table 4:** Risk factors for airflow obstruction in total subjects and in subjects with prior pulmonary tuberculosis.

	FEV_1_/FVC % < 70	FEV_1_/FVC % < 70 and FEV_1_% < 80	FEV_1_/FVC % < 70 and FEV_1_% < 50	FEV_1_/FVC % < 70 and FEV_1_% < 30
**In total subjects** *				
Age (years)	1.089 (1.083–1.095), <0.001	1.061 (1.055–1.068), <0.001	1.060 (1.039–1.082), <0.001	1.048 (0.983–1.117), 0.152
Male sex	3.368 (2.946–3.851), <0.001	2.473 (2.093–2.922), <0.001	2.469 (1.453–4.197), 0.001	9.462 (1.082–82.736), 0.042
Asthma	5.014 (4.022–6.250), <0.001	5.978 (4.753–7.518), <0.001	19.968 (12.862–31.002), <0.001	34.095 (9.215–126.152), <0.001
Smoking amount (pack-year)	1.017 (1.014–1.020), <0.001	1.017 (1.014–1.020), <0.001	1.019 (1.011–1.028), <0.001	1.012 (0.993–1.032), 0.210
Prior pulmonary tuberculosis	2.314 (1.922–2.785), <0.001	2.691 (2.194–3.301), <0.001	4.407 (2.723–7.131), <0.001	8.879 (2.502–31.509), 0.001
**In subjects with normal chest x-rays** *				
Age (years)	1.091 (1.084–1.098), <0.001	1.064 (1.055–1.072), <0.001	1.052 (1.016–1.091), 0.005	-
Male sex	3.715 (3.170–4.353), <0.001	2.809 (2.275–3.470), <0.001	1.628 (0.642–4.131), 0.305	-
Asthma	4.654 (3.584–6.042), <0.001	6.132 (4.607–8.163), <0.001	25.783 (11.997–55.411), <0.001	-
Smoking amount (pack-year)	1.018 (1.014–1.021), <0.001	1.019 (1.015–1.023), <0.001	1.027 (1.011–1.042), 0.001	-
Prior pulmonary tuberculosis	1.308 (0.912–1.876), 0.144	1.589 (1.034–2.444), 0.035	2.299 (0.527–10.021), 0.268	-
**In subjects with prior pulmonary tuberculosis** **				
Age (years)	1.066 (1.048–1.084), <0.001	1.032 (1.014–1.050), 0.001	1.035 (0.988–1.085), 0.142	-
Male sex	1.247 (0.808–1.925), 0.319	0.986 (0.619–1.569), 0.952	1.595 (0.526–4.839), 0.409	-
Asthma	4.622 (2.477–8.627), <0.001	4.467 (2.468–8.086), <0.001	8.722 (3.325–22.878), <0.001	-
Smoking amount (pack-year)	1.027 (1.015–1.039), <0.001	1.017 (1.005–1.028), 0.004	1.011 (0.993–1.029), 0.244	-
Inactive TB lesion on chest X-ray	2.300 (1.606–3.294), <0.001	2.479 (1.656–3.711), <0.001	5.585 (1.263–24.692), 0.023	-

Data are odds ratio (95% confidence interval) and *p* values.

In subjects with normal chest x-rays and with prior pulmonary tuberculosis, there was no patient with FEV_1_/FVC % < 70 and FEV_1_% < 30.

FVC: forced vital capacity; FEV_1_: forced expiratory volume in 1 second; OR: odds ratio; TB: tuberculosis.

*Multivariate logistic regression with age, sex, asthma, smoking amount and prior pulmonary TB.

**Multivariate logistic regression with age, sex, asthma, smoking amount and inactive TB lesion on chest x-ray.

Multivariate regression analysis was performed in subjects with prior pulmonary tuberculosis. Inactive TB lesion on chest x-ray (OR, 2.300; 95% CI, 1.606–3.294), along with age, asthma and smoking amount, was risk factors of airflow obstruction. Inactive TB lesion was significantly related with FEV_1_% < 80 (OR, 2.479; 95% CI, 1.656–3.711) and FEV_1_% < 50 (OR, 5.585; 95% CI, 1.263–24.692) combined with airflow obstruction.

## Discussion

We conducted an analysis of five years of data from the Korean NHANES. The prevalence of prior pulmonary TB was 5.5% in subjects aged ≥ 40 years and pulmonary TB were diagnosed an average of 29 years prior to this study participation. The proportions of airflow obstruction or chronic respiratory symptoms in subjects with prior pulmonary TB were higher than those in subjects without prior pulmonary TB. The impaired pulmonary function was also observed in subjects with no TB sequelae on CXR. Tuberculosis may be associated with permanent pulmonary impairment even in persons with normal chest x-ray.

Important risk factors for airflow obstruction, including COPD, include smoking status, exposure to indoor and outdoor air pollution, occupational hazard, and infection [16,17]. Previous studies have indicated that pulmonary TB can lead to chronic airflow obstruction [4,7,18–20]. The Guangzhou Biobank Cohort Study in china reported that the prevalence of prior pulmonary TB was 24.2%, with an OR of 1.37 for the occurrence of airflow obstruction [4]. In our study, the prevalence of prior pulmonary TB was 5.5%; OR of airflow obstruction in prior pulmonary TB was 2.314. These results suggest that prior pulmonary TB might contribute to the occurrence of chronic airway obstruction in countries with an intermediate burden of TB.

In this study, FVC%, FEV1% and FEV1/FVC% were significantly decreased in those with prior pulmonary TB than in those without prior pulmonary TB. Pulmonary function becomes decreased as the recurrence rate of pulmonary TB increases [8]. A Taiwanese population study reported that risk factors for pulmonary function deterioration are smear-positive disease, initial extensive pulmonary involvement, prolonged antituberculosis treatment, and reduced radiographic improvement after treatment [21]. However, their study had a median follow-up of 16 months, which precluded the evaluation of the long-term effects of TB on pulmonary function. Since pulmonary TB was diagnosed a mean of 29.0 years before participation in the current study, we were able to demonstrate that there is permanent lung function impairment after pulmonary TB. Pasipanodya *et al*. [7] reported that pulmonary function deteriorated after TB development in 71% of non-Hispanic whites, 58% of non-Hispanic blacks, 49% of Asians, and 32% of Hispanics. Our study was conducted in a single race, which excluded racial differences. In this study, the total amount of smoking was higher in subjects with prior pulmonary TB than in those without prior pulmonary TB. Therefore, the comparisons of lung function according to prior pulmonary TB were all adjusted for age, sex, and smoking status. Furthermore, in order to exclude effect of smoking to lung function decline, we performed subgroup analysis in never smoker. Significant lung function decline of prior TB were also observed in never smoker group.

The FEV1% and FEV1/FVC% were significantly decreased in those with prior pulmonary TB than in those without among subjects without abnormalities on CXR although the absolute values of FEV1 were not significantly different. Furthermore, 15.5% of subjects with prior pulmonary TB and no sequelae of pulmonary TB showed airflow obstruction. This data suggests that a history of pulmonary TB might be a predictor of pulmonary function deterioration, despite the absence of pulmonary TB sequelae on CXR. There was no study about pulmonary function impairment in subjects with prior pulmonary TB and no sequelae of pulmonary TB.

In our study, subjects with prior pulmonary TB showed a decrease in FVC% compared with those without prior TB. However, in subjects with normal chest x-ray, there was no difference of FVC% according to prior TB. Pulmonary TB affects FVC through lung tissue scaring, bronchial stenosis, bronchiectasis, and pleural changes [2,22,23]. Pulmonary TB with sequelae can cause restrictive lung disease. In addition, FEF_25-75%_, FEV_6_, and PEF also decreased. One study reported similar results, suggesting that distal airway obstruction can be induced by the sequelae of pulmonary TB [24]. Pulmonary TB may generally affect the lung functions.

In this study, prior pulmonary TB (OR, 2.314), along with age, male, asthma, and smoking mount was risk factor for airflow obstruction. Furthermore, OR of prior pulmonary TB for airflow obstruction were increased according to increment of severity of airflow obstruction. Furthermore, prior pulmonary TB was risk factor for COPD even in subjects with normal chest x-ray. In addition, we found that, in the presence of prior pulmonary TB, inactive TB lesions on CXR were risk factors for occurrence of airflow obstruction (OR, 2.300). Prior TB is a risk factor for airflow obstruction and that the risk is more important when they have inactive lesions on chest X-ray. Therefore, subjects newly diagnosed with pulmonary TB should be meticulously treated to prevent the occurrence of sequelae. Furthermore, subjects with sequelae of pulmonary TB on CXR require early detection and management of impaired pulmonary function, and if they are current smokers, they should be advised to quit smoking. In addition, they should also be given other treatment such as pneumococcal and influenza vaccination.

In a previous study that used data from the 2001 KNHANES, a considerable number of subjects with inactive TB lesions on CXR developed airflow obstruction [5]. However, in this study prior pulmonary TB was defined only as an inactive TB lesion on CXR. Nevertheless, inactive TB lesions on CXR may have been the sequelae of other infectious pulmonary diseases, and subjects with a history of pulmonary TB may present no abnormalities on CXR. Actually, in our study, 36.0% of participants with prior TB history were cured without any sequelae on CXR.

In our study, a past history of physician-diagnosed prior pulmonary TB (mean, 29.0 years prior) was related to impaired pulmonary function, as well as respiratory symptoms, including cough, sputum, and dyspnea. In addition, physical activity limitations due to respiratory symptoms increased by 400% and visual analogue scale scores decreased, indicating a poorer quality of life. In subjects without sequelae of pulmonary TB, cough and physical activity limitations were more frequently observed in subjects with prior TB than those without. One study reported that the quality of life decreased by a mean of 13% after TB treatment [25]. Tuberculosis may be associated with chronic respiratory symptoms and a permanent loss of quality of life.

There were some limitations to this study. First, self-reported data obtained through the questionnaire may present with a recall bias. Second, this study did not have detailed clinical data about bacteriological status of the patients, type and duration of treatment, radiological extension of the disease which can have important influence in results. And discrepancy was observed between total subjects diagnosed with pulmonary TB who received treatment by physicians and subjects with inactive lesions on CXR (5.5% vs. 7.9%). Since pulmonary TB can be spontaneously cured without any treatment [4], the number of subjects with prior pulmonary TB may have been underestimated. In addition, because persons with severe degrees of lung impairment from TB may have already died, pulmonary function impairment is difficult to accurately assess.

## Conclusion

In conclusion, the results of this study suggest that the presence of prior pulmonary TB might be an independent risk factor for airflow obstruction and respiratory symptoms after a significant period following a pulmonary TB infection. And that the risk for airway obstruction is more important when they have inactive lesions on chest X-ray. Considering the significant increase in prevalence of chronic airway disease and burden of medical cost, the spread of TB must be prevented through treatment of latent TB infection and early detection, isolation and treatment of active TB. In addition, subjects with no sequelae of pulmonary TB on CXR can also show impaired pulmonary function, which requires regular follow-ups of lung function for early detection and treatment and smoking cessation.
